# Comparison between simulated annealing algorithms and rapid chain delineation in the construction of genetic maps

**DOI:** 10.1590/S1415-47572010005000033

**Published:** 2010-06-01

**Authors:** Moysés Nascimento, Cosme Damião Cruz, Luiz Alexandre Peternelli, Ana Carolina Mota Campana

**Affiliations:** 1 Departamento de Estatística, Universidade Federal de Viçosa, Viçosa, MGBrazil; 2 Departamento de Biologia Geral, Laboratório de Bioinformática, Universidade Federal de Viçosa, Viçosa, MGBrazil

**Keywords:** better order, genetic mapping, genomic analyses, stochastic optimization

## Abstract

The efficiency of simulated annealing algorithms and rapid chain delineation in establishing the best linkage order, when constructing genetic maps, was evaluated. Linkage refers to the phenomenon by which two or more genes, or even more molecular markers, can be present in the same chromosome or linkage group. In order to evaluate the capacity of algorithms, four F_2_ co-dominant populations, 50, 100, 200 and 1000 in size, were simulated. For each population, a genome with four linkage groups (100 cM) was generated. The linkage groups possessed 51, 21, 11 and 6 marks, respectively, and a corresponding distance of 2, 5, 10 and 20 cM between adjacent marks, thereby causing various degrees of saturation. For very saturated groups, with an adjacent distance between marks of 2 cM and in greater number, *i.e.*, 51, the method based upon stochastic simulation by simulated annealing presented orders with distances equivalent to or lower than rapid chain delineation. Otherwise, the two methods were commensurate through presenting the same SARF distance*.*

## Introduction

Genetic mapping favors breeding activities, by associating one or more marks to those genes of economic interest and/or control quantitative characteristics (QTL), with a reasonable chance of use in assisted selection, hence the extreme importance of the precise construction of genetic maps in the successful introduction of strategies in breeding programs.

One of the most important stages in the construction of linkage maps is the ordering of the genetic markers within each linkage group (Mollinari *et al.*, 2008). It is said that two or more genes, or molecular markers, are connected if they belong to the same chromosome or linkage group.

Several methods for ordering markers are mentioned in the literature, such as rapid chain delineation (Doerge, 1996), seriation (Buetow and Chakravarti, 1987a,b), simulated annealing (Kirkpatrick *et al.*, 1983) and branch and bound (Thompson, 1987). Rapid chain delineation consists of obtaining a preliminary order for loci based upon a recombination matrix of all the pairs of marks. Successive inversions are then attempted with triple marks, in order to minimize the sum of adjacent recombination fractions (SARF). Seriation is a simple method, in which a set of rules is proposed, based upon the recombination fractions between two loci (Liu, 1998). The method of branch and bound is based on a tree structure, a recombinant number being calculated for each branch. Simulated annealing, a stochastic simulation method, corresponds to the famous MCMC method (Markov Chain Monte Carlo, specifically the Metropolis-Hastings Algorithm), modified in such a way as to become an optimization algorithm. In order to arrive at an ordering solution through these methods, several criteria may be used, namely the minimum Sum of Adjacent Recombination Fractions (SARF) (Falk, 1992), the minimum Product of Adjacent Recombination Fractions (PARF) (Wilson, 1988), and the maximum Sum of Adjacent LOD Scores (SALOD) (Weeks and Lange, 1987).

Several studies using genetic mapping as a basis for breeding are to be found in the literature. The study of Silva *et al.* (2008) intended to map and detect QTLs in chromosome 4 of swine, and associate these with the carcass and characteristics of internal organs in an F2 population. Miyata *et al.* (2007) investigated the presence of QTLs in BTA14 chromosomes, by weight at birth and after 60 days, also in an F2 experimental station. Soares *et al.* (2008) also aimed to detect QTLs related to protein content in soybean cultivated in two divergent tropical environments, thereby constructing a genetic map of genotypes adapted to tropical conditions.

In spite of the outstanding significance of ordering markers when constructing linkage maps, and of the numerous methods designed to provide solutions for the problem of ordering itself, it is difficult to find works which present comparative analyses of these methods. Mollinari *et al.* (2008) compared the rapid chain delineation and seriation methods, and concluded that final results were alike.

Thus, the aim hereby was to evaluate the efficacy of both the simulated annealing and rapid chain delineation methods, in establishing the most efficient linkage order when constructing genetic maps. The study was so developed as to capacitate its competent reproduction and use in research. The problem of mark ordering is described as the problem of the traveling salesman.

## Material and Methods

In order to create a real situation and compare the efficiency of the methods, four F_2_ co-dominant populations in various sizes (50, 100, 200 and 1000) were simulated. Genomes were generated for each population, with four linkage groups, each 100 cM in size. There were 51, 21, 11 and 6 marks in each linkage group, with distances of 2, 5, 10 and 20 cM, respectively, between adjacent marks, thus causing various degrees of saturation. The groups were composed of:

• First linkage group: marker 1 (*m*_1_), marker 2 (*m*_2_), ..., marker 51 (*m*_51_), with intervals between adjacent marks of 2 cM;

• Second linkage group: marker 52 (*m*_52_), marker 53 (*m*_53_),..., marker 72 (*m*_72_), with intervals between adjacent marks of 5 cM;

• Third linkage group: marker 73 (*m*_73_), marker 74 (*m*_74_),..., marker 83 (*m*_83_), with intervals between adjacent marks of 10 cM;

• Fourth linkage group: marker 84 (*m*_84_), marker 85 (*m*_85_),..., marker 89 (*m*_89_), with intervals between adjacent marks of 20 cM.

The “Complex Genome Simulation” module GQMOL (Cruz, 2007) for computing application was used in obtaining the above populations.

The problem of mark ordering by performing the analogies necessary for solving the traveling salesman problem, can be described in the following way: let *I* = {1, ..., *k*} be a set of indices and M = {*m*_*i*_: *i* ∈ *I*} a set of markers indexed by *i*. Consider that *D*_*ij*_ represents the distance between the marker *m*_*i*_ and the marker *m*_*j*_ and define Λ as a set of all the possible permutations of the elements of the *M* set. An *M* element will be denoted by 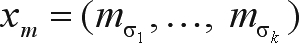, where (σ_*i*_, ..., σ_*k*_) is a permutation of the elements of set *I*. A permutation *x*_*m*_ ∈ Λ can be understood as an order to by-pass all the markers. The problem is to find an order that minimizes the distance necessary to by-pass all the markers only once, without the need of returning to the origin.

Let *f*(*x*_*m*_) be the function that associates SARF, or the total distance covered, to each order *x*_*m*_ ∈ Λ, or, in other words, 
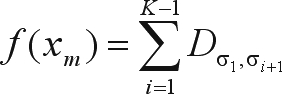
, where 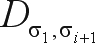 is the distance between the marks 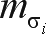 and 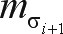. The objective is to find the *x*_*m*_ ∈ Λ order that minimizes *f*(*x*_*m*_). Simulated annealing and rapid chain delineation algorithms were used for obtaining a numeric approximation for the solution of this problem.

Simulated annealing is a small modification in the famous MCMC algorithm of Metropolis-Hastings (Hastings, 1970), thereby transforming it into an optimization algorithm, known as simulated annealing (Kirkpatrick *et al.*, 1983). The main idea inherent in this method is borrowed from physics. In condensed matter physics, annealing is the thermic process used to minimize the free energy of a solid. Informally, the process may be described as occurring in two stages: (i) an increase in temperature to melting; (ii) followed by a slow decrease in temperature until particle re-organization in a state of minimum energy. This physical process may be simulated computationally by using the Metropolis-Hastings algorithm.

Suppose that the current state of the solid is *x* and that the energy of this state is *H*(*x*). A candidate state *y* of energy *H*(*y*) is generated by applying slight perturbation to state *x*. The following probability is used in the decision-rule for accepting the candidate state:



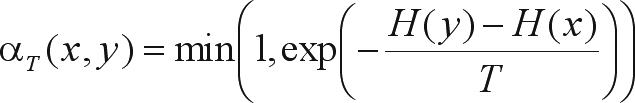
,

with *T* indicating temperature. If cooling is slow, the solid reaches thermic balance at each temperature. From the point of view ‘simulation', this means generating several transactions at a certain temperature *T* (Robert and Casella, 2004).

For the problem of marker ordering, there is the following analogy:

• The solutions of problem ordering (optimization), or, in other words, the elements *x*_*m*_ ∈ Λ, are equivalent to the physical states of *x*;

• The function 
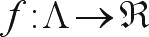
 (SARF) is equivalent to the function ‘soil energy', *H*(*x*);

• A candidate order *y*_*m*_ of distance given by 
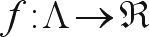
 is equivalent to a candidate state *y* of energy *H*(*y*);

• A control parameter *c* > 0 is equivalent to the temperature.

Let 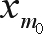 be an initial order, *c*_0_ the initial control parameter and *L*_0_ the initial number of iterations used for an equal value of *c*_0_. Simulated annealing can thus be described in the following manner:

1) Choose *n* = 0, 
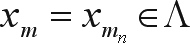
, *c*_0_ and *L*_0_;

2) Make *i* vary from 1 to *L*_*n*_

• Generate *y*_*m*_ in the neighborhood of *x*_*m*_ and generate a random variable *X* ~ *U*(0, 1);

• If *f*(*y*_*m*_) ≤ *f*(*x*_*m*_), then *x*_*m*_ ← *y*_*m*_;

• If *f*(*y*_*m*_) > *f*(*x*_*m*_) and 
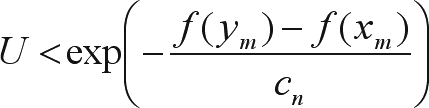
, then *x*_*m*_ ← *y*_*m*_;

• End of operation;

*n* ← *n* + 1

Define *c*_*n*_ and *L*_*n*_, and return to step 2 until the ‘stop' criterion, where *L*_*n*_ is the number of chain transactions in each temperature (*c*_*n*_).

The rapid chain delineation algorithm (Doerge, 1996) constitutes a simple way of molecular marker ordering within linkage groups. This algorithm can be described in the following manner:

1) Verify for which pairs of markers (*m*_*i*_, *m*_*j*_) the estimate of recombination fractions between pairs is the lowest. These markers will start the chain;

2) Verify which is the unmapped marker (*m*_*k*_) presenting the lowest estimate of recombination fractions with one of the terminal markers. Place the two together accordingly;

3) Repeat the procedure until all the markers are added to the chain;

4) Then, attempt successive inversions in double and triple marks, in order to minimize SARF (the sum of adjacent recombination fractions).

One hundred repetitions were carried out with the stochastic simulation algorithm, simulated annealing, and the results compared to those provided by the rapid chain delineation method. The criterion used for reaching this solution was minimum SARF.

## Results and Discussion

**Figure 1:**
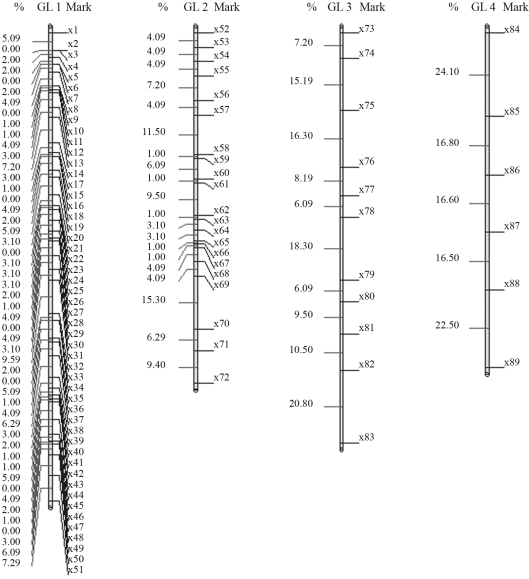
Solutions for mark ordering through rapid chain delineation (obtained using GQMOL software) for a population of 50 individuals.

The results obtained with GQMOL software, which finds the solution for the problem through the rapid chain delineation method, are presented in Figures 1 and 4. For numeric approximation of the solution to the marker ordering problem, when using the simulated annealing algorithm, it is necessary to define a neighborhood system in Λ, or, in other words, a candidate permutation of markers. A system was adopted in which the typical neighbor (candidate order) of an order



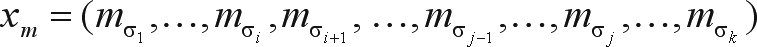


was defined as



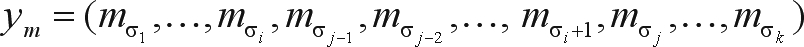
.

During the application of the algorithm, it was defined to uniformly choose an order *y*_*m*_ in the set of possible orders. The algorithm was implemented in the *R* version 2.7.1 programming language (R DEVELOPMENT CORE TEAM, 2007). An Intel Core 2 Duo T5750 2.0 GHz processor was used with a 3 Gb RAM memory, Windows XP SP2.

The parameter of control in the *n*^th^ algorithm iteration, denoted by *c*_*n*_, was calculated based upon the expression



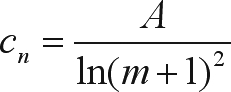
,

where *m* is the number of iterations of the algorithm and *A* is a constant chosen in a convenient form, described as follows:

The choice of *A* is undertaken in such a way that the simulated annealing algorithm escapes from the minimum places of interest function (SARF) to reach the global minimum. Therefore, constant A must be chosen in such a way that all the initial orders are accepted. In the present case, 2 was considered as the value of this constant.

One hundred repetitions were carried out, with a comparison of the best result from simulated annealing to that from the rapid chain delineation method.

The final results through simulated annealing for a population composed of 50 individuals in linkage group 1, and as a numeric solution, is given by the following order, *m*_3_, *m*_2_, *m*_4_, *m*_5_, ..., *m*_14_, *m*_15_, *m*_17_, *m*_16_, *m*_18_, *m*_19_, ..., *m*_32_, *m*_34_, *m*_33_, *m*_1_, *m*_35_, *m*_36_, ..., *m*_49_, *m*_50_, *m*_51_, with a total SARF distance of 129,90 cM, thus being of smaller size than the 135,00 cM from rapid chain delineation (Figure 1). For the second, third and fourth linkage groups, the solutions obtained through simulated annealing are the same as those by the method implemented in the GQMOL program, also apparent in Figure 1, with distances of 101,10 , 118,20 and 96,50, respectively. Figure 5 shows the evolution of total distances of algorithmic iteration in each of the linkage groups analyzed.

**Figure 2:**
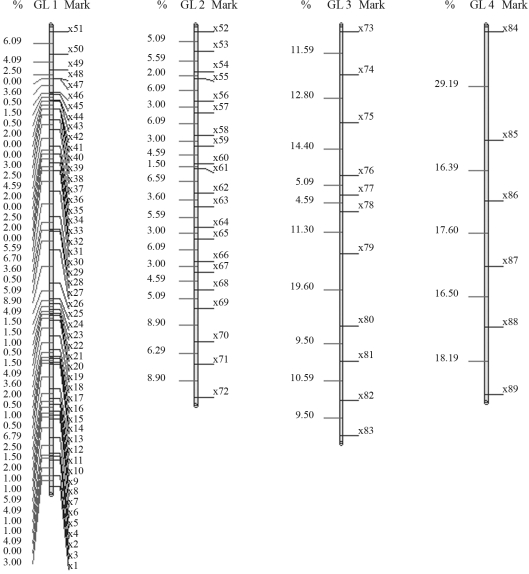
Solution obtained for mark ordering through rapid chain delineation (obtained using GQMOL software) for a population of 100 individuals.

For a population with 100 individuals, the solution obtained for the first linkage group is given by the following order: *m*_51_, *m*_50_, ..., *m*_22_, *m*_21_, *m*_19_, *m*_20_, *m*_1_, *m*_18_, *m*_17_, ..., *m*_3_, *m*_2_. In this order, SARF is 117,60 cM. On comparing this specific solution with that from rapid chain delineation (Figure 2), it can be seen that the total distance is shorter in the former than in the latter method (122,70 cM). The solutions obtained for linkage groups 2, 3 and 4 by simulated annealing are the same as those found by way of the rapid chain delineation method, with a total distance of 98,70, 109,00 and 97,90 cM, respectively. These orders are presented in Figure 2. Figure 6 shows the evolution of total algorithmic iteration distances in each of the linkage groups analyzed.

**Figure 3:**
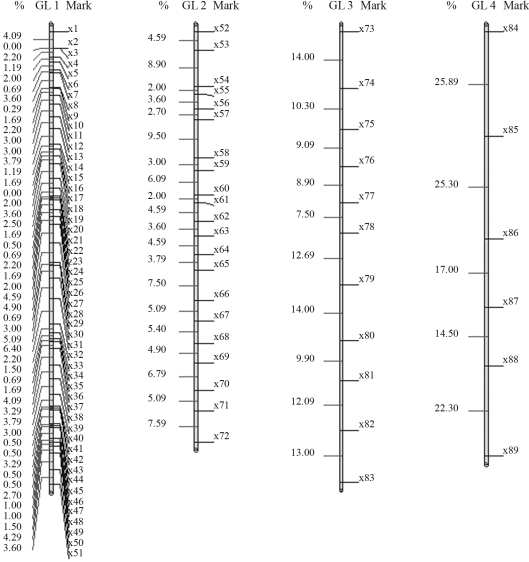
Solution obtained for mark ordering through rapid chain delineation (obtained using GQMOL software) for a population of 200 individuals.

On considering a population of 200 individuals, the numeric solution for the first linkage group, when employing stochastic optimization, is given by the following order: *m*_51_, *m*_50_, ..., *m*_46_, *m*_45_, *m*_43_, *m*_44_, *m*_42_, *m*_41_, ..., *m*_20_, *m*_19_, *m*_1_, *m*_18_, *m*_16_, *m*_17_, *m*_15_, *m*_14_, ..., *m*_2_, *m*_3_, with a total distance of 108,40 cM, thus smaller than that provided by the method implemented in the GQMOL program, whereby the SARF value was 112,00 cM. The corresponding numeric order is presented in Figure 3. As regards the three remaining linkage groups, the solutions arrived at by both methods are identical, and are also perceptible in Figure 3. These orders presented total distances of 101,40, 111,50 and 105,00 cM, respectively. The evolution of total distances of algorithmic iterations in each linkage group analyzed can be seen in Figure 7.

According to Ferreira *et al.* (2006), a total of 200 individuals is considered large enough for constructing reasonably precise genetic maps. They evaluated F_2_ populations with dominant and co-dominant markers, backcrossing, recombinant inbred lines (RIL) and double-haploid. Nevertheless, on comparison, algorithmic performance in simulated annealing was superior to that in rapid chain delineation, even with sufficiently large populations.

**Figure 4:**
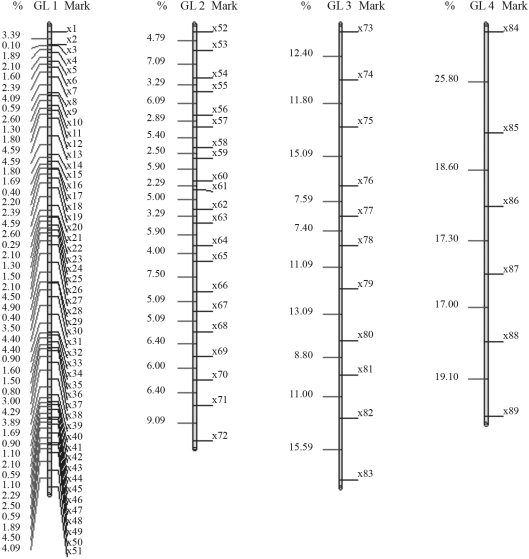
Solution obtained for mark ordering through rapid chain delineation (obtained with GQMOL software) for a population of 1000 individuals

The analysis of a population of 1000 individuals revealed that the order established by the rapid chain delineation method was identical to that from a population of 200 individuals, thus corroborating the results by Ferreira *et al.*, (2006). Nevertheless, application of the algorithm of simulated annealing gave rise to the following order as a numeric solution: *m*_51_, *m*_50_, ..., *m*_19_, *m*_1_, *m*_18_, *m*_17_, ..., *m*_3_, *m*_2_. The total distance was 112,30 cM, thus shorter than that arising from the other method evaluated (*SARF*) of 115,60 cM. The numeric order appears in Figure 4. The solutions found in the other linkage groups are mutually equivalent (Figure 4).

The total distances for these orders are 104,10, 113,90 and 97,80 cM, for the second, third and fourth linkage groups, respectively. The evolution of the total distances of algorithmic iteration in the linkage groups was analyzed (Figure 8).

In all the cases studied, execution of simulated annealing took less than 131 s, at the most (Table 1). As rapid chain delineation is a deterministic method, no repetitions were used, the time-span not exceeding 5 s in the various cases studied. The percentage of times, in 100 repetitions, that results from simulated annealing were higher (lowest SARF value) than those from rapid chain delineation, are presented in Table 1. As can be observed, in the first linkage group of each population, results from simulated annealing were higher in less than 50% of the cases, although there were orders with a lower SARF value in the same groups.

**Figure 5:**
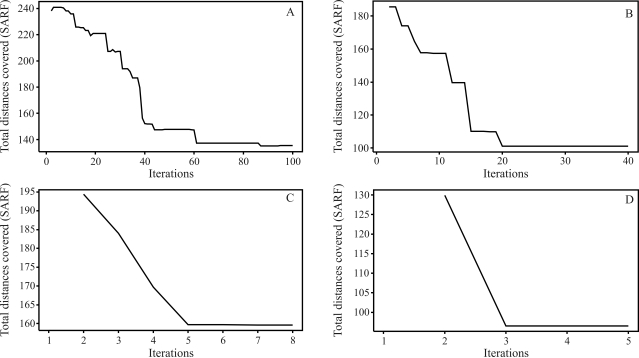
Evolution of the total distances at each algorithm iteration in each population of 50 individuals. (A) linkage group 1 (B) linkage group 2 (C) linkage group 3 (D) linkage group 4.

Figures 5 and 8 demonstrate that the number of necessary iterations for the algorithm to obtain a satisfactory result depends on the number of markers in the study, since the higher the number of marks in the linkage group, the higher the number of iterations.

It is obvious from the data that, in the case of the most saturated linkage groups, namely those with shorter distances between adjacent marks, viz., 2 cM, achievements through simulated annealing were similar or better than those by rapid chain delineation in less than 50% of the repetitions. Nevertheless, on considering the criterion used for constructing linkage maps, *i.e.* the lowest SARF value, the former proved to be more efficient. Such a superior performance can also be explained by the number of markers, for, as the algorithm in question is stochastic, the higher the number of markers, the more efficient the method when compared to rapid chain delineation, ultimately leading to the possibility of analyzing a higher number of possible orders, as occurred here. As to the other linkage groups, with lower saturation levels and consequently less markers, results were similar with the two methods.

Furthermore, the number of individuals constituting the population has no effect on results when using the algorithm, since recombination frequencies, previously calculated for each pair of markers, are fundamental when ordering. So, the number of individuals exerts an influence only on the precision of estimates, but not on the ordering, thereby possibly leading to the construction of imprecise linkage maps. According to Mollinari *et al.*, (2008), it was concluded that the rapid chain delineation and the seriation methods are both equivalent, whereby it is possible to infer that simulated annealing is also superior to the seriation method in certain situations.

## Conclusions

In the present study, simulated annealing and rapid chain delineation algorithms were compared when establishing the best linkage order in the construction of genetic maps, in populations of different sizes and saturation levels. It was observed that, for very saturated linkage groups, with an adjacent distance between marks of 2 cM, and a higher number of marks, *e.g.* 51, the method based on stochastic simulation, viz., simulated annealing, presented orders with distances (SARF) equal to or shorter than rapid chain delineation in less than 50% of the repetitions. Nevertheless, the former method appears to be more interesting than the latter in these cases, as the criterion used for constructing linkage maps is to take into consideration the order of markers with lower SARF values. In the other cases, the two methods were alike, presenting the same SARF distances. Furthermore, it was noted that the number of individuals in the population does not affect ordering, although it does affect the estimates of recombination frequencies. The average time taken for simulated annealing execution did not exceed 112 s, thus not an obstacle for implementation.

The data from the present work demonstrate the relevance of the method used for ordering markers in the construction of genetic maps. Therefore, future studies should be carried out, in order to evaluate all the methods encountered in the literature, and thus facilitate their use according to the situation.

**Figure 6:**
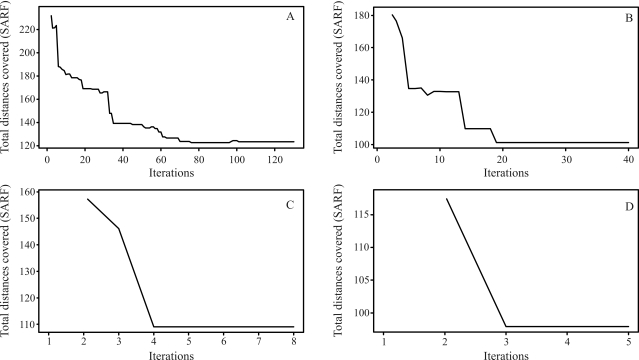
Evolution of the total distances at each algorithm iteration, in each population of 100 individuals. (A) linkage group 1 (B) linkage group 2 (C) linkage group 3 (D) linkage group 4.

**Figure 7:**
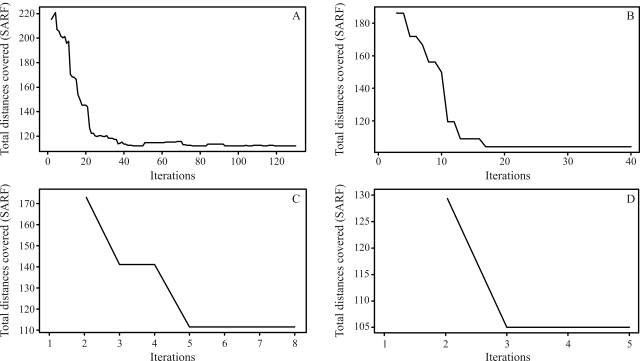
Evolution of total distances at each algorithm iteration in each population of 200 individuals. (A) linkage group 1 (B) linkage group 2 (C) linkage group 3 (D) linkage group 4.

**Figure 8:**
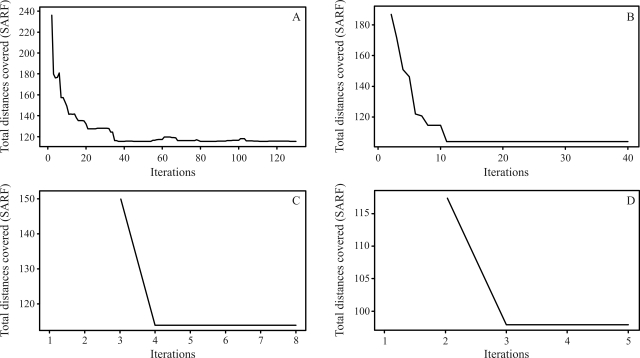
Evolution of the total distances at each algorithm iteration in each population of 1000 individuals. (A) linkage group 1 (B) linkage group 2 (C) linkage group 3 (D) linkage group 4.

## Figures and Tables

**Table 1:** The average time spent on simulated annealing (S.A.), and the percentage of times when the results were higher than those from rapid chain delineation in 100 repetitions.

Parameter	Algorithm	Size of the population																		
		50					100					200					1000			
		Linkage Groups																		
		1	2	3	4		1	2	3	4		1	2	3	4		1	2	3	4
Percentage (%)	S. A.	30	100	100	100		35	100	100	100		8	100	100	100		30	100	100	100
Time (s)		73	3	2	< 1		105	15	3	< 1		112	14	1	< 1		131	17	2	< 1
